# Arthropod-Borne Flaviviruses in Pregnancy

**DOI:** 10.3390/microorganisms11020433

**Published:** 2023-02-08

**Authors:** Annaleise R. Howard-Jones, David Pham, Rebecca Sparks, Susan Maddocks, Dominic E. Dwyer, Jen Kok, Kerri Basile

**Affiliations:** 1 Centre for Infectious Diseases and Microbiology Laboratory Services, Institute of Clinical Pathology and Medical Research—NSW Health Pathology, Westmead, NSW 2145, Australia; 2 Sydney Infectious Diseases Institute, The University of Sydney, Westmead, NSW 2145, Australia; 3 Faculty of Medicine and Health, University of Sydney, Camperdown, NSW 2006, Australia; 4 Centre for Infectious Diseases and Microbiology-Public Health, Westmead, NSW 2145, Australia

**Keywords:** flavivirus, pregnancy, neonate, Zika virus, Dengue virus, West Nile virus, Japanese encephalitis virus, Yellow fever virus

## Abstract

Flaviviruses are a diverse group of enveloped RNA viruses that cause significant clinical manifestations in the pregnancy and postpartum periods. This review highlights the epidemiology, pathophysiology, clinical features, diagnosis, and prevention of the key arthropod-borne flaviviruses of concern in pregnancy and the neonatal period—Zika, Dengue, Japanese encephalitis, West Nile, and Yellow fever viruses. Increased disease severity during pregnancy, risk of congenital malformations, and manifestations of postnatal infection vary widely amongst this virus family and may be quite marked. Laboratory confirmation of infection is complex, especially due to the reliance on serology for which flavivirus cross-reactivity challenges diagnostic specificity. As such, a thorough clinical history including relevant geographic exposures and prior vaccinations is paramount for accurate diagnosis. Novel vaccines are eagerly anticipated to ameliorate the impact of these flaviviruses, particularly neuroinvasive disease manifestations and congenital infection, with consideration of vaccine safety in pregnant women and children pivotal. Moving forward, the geographical spread of flaviviruses, as for other zoonoses, will be heavily influenced by climate change due to the potential expansion of vector and reservoir host habitats. Ongoing ‘One Health’ engagement across the human-animal-environment interface is critical to detect and responding to emergent flavivirus epidemics.

## 1. Introduction

**Figure 1:**
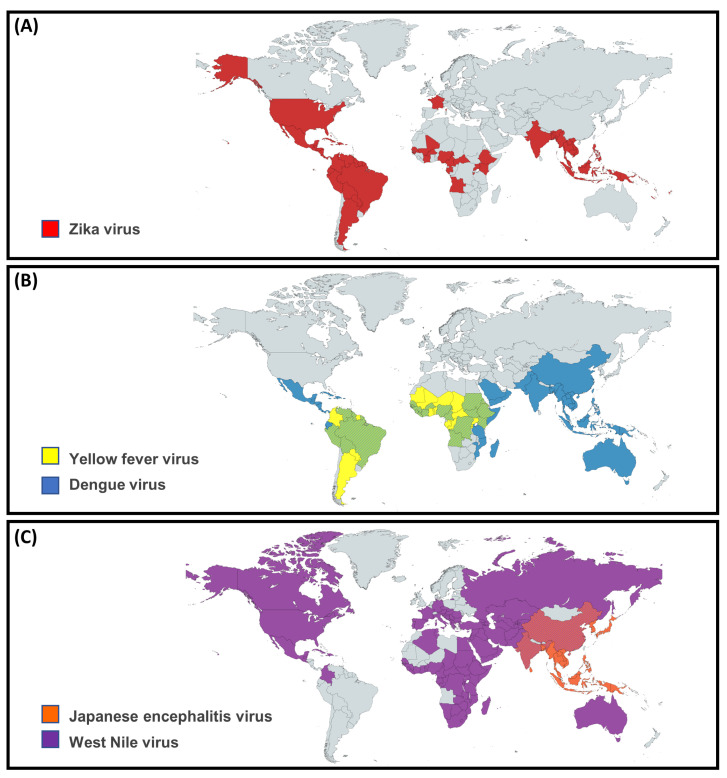
Global distribution of flaviviruses of importance in pregnancy including (**A**) Zika virus; (**B**) Yellow fever virus and dengue virus; and (**C**) Japanese encephalitis virus and West Nile virus [1,3,4]. Of note, the potential endemicity of JEV in Australia is currently under investigation in light of a recent outbreak in 2022 [5].

Flaviviruses are important human pathogens that comprise a broad group of enveloped positive-stranded RNA viruses with over 75 defined species [1]. Flaviviruses naturally circulate in enzootic interactions between their reservoir hosts and arthropod vectors (primarily mosquitoes), and are responsible for sporadic epidemics as well as established endemicity in certain geographic regions (Figure 1) [2]. The main flaviviruses of concern during pregnancy are Dengue virus (DENV), Zika virus (ZIKV), Japanese encephalitis virus (JEV), West Nile virus (WNV), and Yellow fever virus (YFV). Many of these viruses are neurotropic and/or have marked impacts on the developing fetus. ZIKV in particular is associated with a profound congenital infection syndrome which manifests with neonatal microcephaly and severe long-term neurodevelopmental consequences.

Between 50–80% of human flavivirus infections are asymptomatic [6,7]. Only a minority of symptomatic cases present with neuroinvasive disease, although mortality and long-term morbidity are significant following clinically apparent neuroinvasive illness [8]. There are no established risk factors or biomarkers that can reliably predict who will develop neuroinvasive disease, however, host factors (such as age, prior infection, and immunodeficiency) are almost certainly as important as viral and environmental features [9].

**Figure 2:**
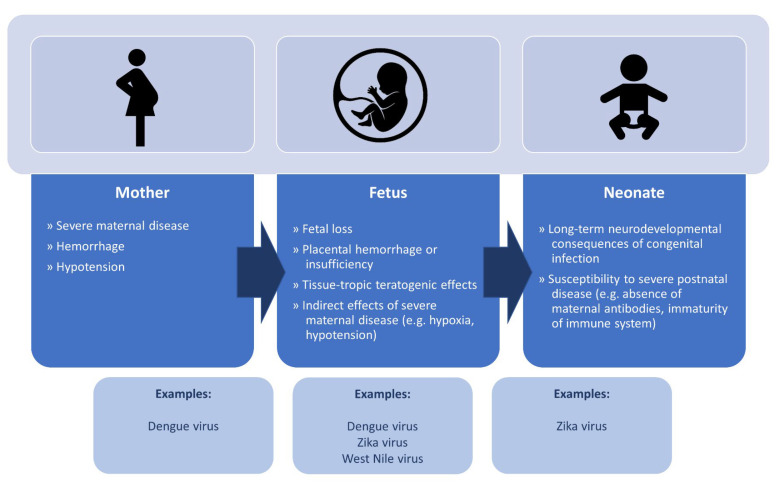
Pathophysiological impacts of flavivirus infection on the pregnant mother, fetus, and neonate.

Pregnancy represents a known risk for disease with certain flaviviruses [10]. In the antenatal and postnatal periods, flavivirus infection can have substantive impacts on the pregnant woman (due to increased risk of severe disease), the developing fetus (due to tissue-specific teratogenic effects at different developmental stages, placental insufficiency or hemorrhage, or indirectly due to severe maternal disease) or the neonate in the context of postnatal acquisition of infection (Figure 2) [10]. The spectrum of congenital disease varies depending on the particular flavivirus, due largely to their differential ability to cross the placenta and their particular tissue tropism.

As a viral family, the flaviviruses confer clinical syndromes with a high degree of overlap with one another and also with other viral, bacterial, and parasitic infections which challenges diagnostic efforts. In an acute encephalitic or non-encephalitic illness, alternative pathogens to the flaviviruses such as alphaviruses (for example, Chikungunya), parvovirus B19, rubella, other respiratory viruses (such as enterovirus or adenovirus), measles, leptospirosis, rickettsial infection, Group A *Streptococcus,* and malaria must be considered.

The diagnosis of flavivirus infections is nuanced. Critical to the accurate diagnosis is a thorough clinical history including symptoms, pregnancy status, immune function, travel history (including any recent sexual partners), vaccination history as well as any exposures to potential arthropod vectors. These details along with clinical examination findings must be integrated with knowledge of the virus’ geographic distribution (including endemic regions, contemporaneous epidemics, and emergence in new locations), and arthropod activity and characterization to ascertain likely differential diagnoses to tailor investigations and empiric treatment. Laboratory diagnoses rely on both serology and molecular detection. Serological testing is prone to antibody cross-reactions amongst closely related viral epitopes, so specificity is improved by testing patients’ sera against a panel of relevant flaviviruses, with quantitation of virus-specific IgG and IgM titers. Molecular diagnosis using nucleic acid amplification testing (NAAT), although specific, is limited by a short window of viremia and hence a requirement for early targeted sampling. As a result, serological assays often have higher utility than molecular testing in ascertaining the extent of an outbreak. Viral serotyping, genotyping, and isolation are useful for further characterization. 

This review focuses on the globally important arthropod-associated flaviviruses linked to significant impacts in the pregnancy and post-partum periods, including ZIKV, DENV, JEV, WNV and YFV. We discuss key features of epidemiology, pathophysiology, clinical features, diagnosis, and prevention of flaviviruses in pregnancy, and the commonalities between the flaviviruses, as well as highlighting important areas for future research. The trivalent lens on maternal, fetal, and neonatal disease (Figure 2) is critical since the manifestations of these viruses are at times quite disparate across these patient subgroups due to variable pathogenesis, trans-placental transmission rates and tissue tropism.

## 2. Overarching Pathophysiology

Across the severity spectrum of flavivirus infections, significant sequelae are typically associated with their neuroinvasive manifestations. The exact pathophysiology underpinning flavivirus neuroinvasion remains poorly characterized [11] and there is some variability in tropism. JEV and WNV have a predilection for the central nervous system (CNS), whilst ZIKV shows relative sparing of the mature CNS in adults and instead targets the peripheral nervous system of adults causing Guillain-Barré syndrome (GBS).

Apoptosis of neuronal cells and glial cell damage with scarring are seen in neuroinvasive diseases [11]. Reduced integrity of the blood-brain barrier through cytokine-mediated inflammation or retrograde axonal transport have been proposed as mechanisms, but firm evidence is still awaited [11]. DENV, WNV, and ZIKV have been demonstrated to use cell membrane lipid rafts as a means of viral entry to the cell [12,13]. Once attached to the cell, the virus is taken up by endocytic vesicles, with membrane fusion promoted in the low pH environment of the endosome [1]. Replication of endoplasmic reticulum-derived membranous structures is followed by viral maturation and release.

Despite the sparing of CNS invasion in adults, ZIKV in pregnancy causes marked destruction of the neural progenitor cells of the developing fetus and has a tropism for placental cells, phenomena not commonly seen with other flaviviruses (such as WNV and JEV). Severe congenital CNS abnormalities in the developing fetus are the clinical corollary. The tropism of ZIKV for placental cells occurs via infection of placental stromal macrophages (Hofbauer cells) as well as early trophoblasts [11], which likely underpins its mode of vertical transmission. Interestingly, WNV has been shown to invade neural progenitor cells in murine fetuses and to infect placental tissue ex vivo [14] but the clinical correlation in human pregnancies is rare. The reason for the unique profile of ZIKV and its propensity to cause congenital infection is unclear however may rest on the more limited trans-placental passage of the other flaviviruses, or on differential targeting of cellular entry mechanisms [11].

## 3. Zika Virus

### 3.1. Epidemiology

ZIKV was first identified in monkeys in the Zika forest in Uganda in 1947, with the first human case diagnosed in 1952. It is now endemic across South and Central America, South, and South-East Asia, the Western Pacific, Africa, and France with many major outbreaks reported [15] (Figure 1A). Occasional cases have been reported in the southern states and territories of the United States of America including Florida, Texas and Puerto Rico.

### 3.2. Pathogenesis

The primary mode of ZIKV acquisition is through the bite of the *Aedes aegypti* mosquito [16], or through vertical transmission from an infected mother. Although the virus has been detected in breast milk of infected mothers, clinical data demonstrating transmission via breast milk to the infant is lacking [17]. Sexual transmission [18] and acquisition via solid organ or blood transfusions are well-described [19]. 

The neuroinvasion by ZIKV in the developing fetus leads to the well described clinical sequelae of microcephaly and neurodevelopmental compromise. Placental insufficiency also results in downstream effects on fetal growth and development independent of the direct neurological insult [10].

### 3.3. Clinical Features

The incubation period for ZIKV is between two and 14 days, and the disease spectrum is very broad (Table 1). Three-quarters of infected persons will remain asymptomatic. In those with clinical disease, illness typically lasts two to seven days and manifests as fever with a pruritic maculopapular rash (involving the palms and soles), arthralgia, headache, myalgia, conjunctival hyperemia and lethargy [20]. Relapses are well described [21]. GBS is a significant and well-described complication [22]; meningoencephalitis and transverse myelitis are rare complications [23].

#### 3.3.1. Pregnant Women

The clinical manifestations of ZIKV in pregnant women are similar to non-pregnant adults, with 20–25% showing symptomatic disease [24]. A single case of GBS in pregnancy has been reported [25]; meningoencephalitis and transverse myelitis have not been described in the pregnant population thus far.

#### 3.3.2. Fetus

ZIKV is one of the most devastating congenital infections with a vertical transmission rate reported between 26–65% with severe fetal sequelae [26,27]. ZIKV infection is associated with a high rate of fetal loss (7–14%) encompassing mothers with asymptomatic as well as symptomatic infection [25,27]. Ventriculomegaly (33%), microcephaly (24%), intracranial calcifications (27%), ophthalmological anomalies and neurodevelopmental abnormalities are the most commonly described sequelae [28,29,30]. Adverse outcomes are more common with maternal infection in the first (55%) and second (52%) trimester but are also seen in the third trimester (29%) [25]. 

#### 3.3.3. Neonate

Congenital Zika syndrome (CZS) is well described and comprises often-severe microcephaly, hypertonia or hyperreflexia, seizures, irritability, arthrogryposis, ocular abnormalities (macular scarring or focal retinal mottling), and sensorineural hearing loss [31]. CZS is significantly associated with a risk of long-term neurodevelopmental consequences out to at least three years of age [32,33]. Suspected or confirmed maternal ZIKV infection during pregnancy or signs of possible CZS should prompt neonatal testing with guidance from microbiologists, infectious diseases, and neonatal specialists, and/or expert guidelines [34]. Neonatal whole blood (or serum) and urine should be tested for ZIKV RNA using NAAT and serum for ZIKV-specific IgM. Placental histopathology (ideally with NAAT for ZIKV RNA and immunohistochemical staining) should also be performed if available. 

Postnatal acquisition of ZIKV in children gives a similar clinical spectrum to adults, though neonate-specific data is lacking. Data from non-human primates suggest that maternally derived antibodies may persist for 3–5 months [35], consistent with known postnatal patterns of maternal antibodies [36,37]. Long-term developmental complications have been observed in 45% of children with postnatal ZIKV meningoencephalitis [38], but outcomes of infection in the neonatal period specifically have not been described.

## 4. Dengue Virus

### 4.1. Epidemiology

DENV is one of the most common vector-borne diseases worldwide with an estimated 390 million infections annually, primarily reported in tropical and subtropical regions (Figure 1A) [39]. With ongoing climate change, international travel, urban population growth, and evolving interactions between mosquitos and humans, the geographic distribution and incidence of dengue fever has increased dramatically over the past century [40]. There are four DENV serotypes, each of which is further subdivided into multiple genotypes [41,42,43].

### 4.2. Pathogenesis

Humans and primates are the primary hosts of DENV, and transmission is predominantly via *Aedes* spp. mosquitos [44]. The incubation period after a mosquito bite is 3–7 days [43]. DENV replicates in local tissues, and then disseminates into the bloodstream [45]. Viremia typically lasts 3–7 days and is sufficiently high that an uninfected mosquito can acquire DENV from a human host during this period. 

DENV infection typically confers life-long immunity to re-infection with the same serotype. However, immunity to the other serotypes is transient, and a severe ‘secondary infection’ syndrome is well described following infection with a different DENV serotype. This phenomenon is thought to occur due to antibody-dependent enhancement, formation of immune complexes, and/or accelerated T-lymphocyte inflammatory responses [46,47,48,49]. Tertiary or quaternary infections are less common [50].

### 4.3. Clinical Features

Most DENV infections are asymptomatic or cause a mild non-specific febrile illness (Table 1). However, dengue hemorrhagic fever (DHF) or dengue shock syndrome (DSS) can be life-threatening manifestations, and occur in about 1% of DENV infections [51], with the risk significantly higher in secondary infections. The pathogenesis of DHF/DSS involves blood dyscrasias due to the effect of DENV on bone marrow, and capillary leak due to endothelial cell dysfunction [52,53].

#### 4.3.1. Pregnant Women

DENV infection is associated with more severe disease and higher mortality in pregnant women compared to the broader population (odds ratio for maternal mortality (OR) 4.14, 95% CI 1.17-14.73) [54,55]. As early features of dengue can mimic physiological changes observed in normal pregnancy or laboratory abnormalities of the hemolysis, elevated liver enzymes, and low platelets (HELLP syndrome of pregnancy), delayed recognition and treatment of severe dengue can contribute to poor maternal outcomes. Severe thrombocytopenia or DHF can result in post-partum hemorrhage following either vaginal delivery or lower segment Caesarean section [56,57]. 

#### 4.3.2. Fetus

DENV infection during pregnancy is associated with higher risk of prematurity (OR 1.71, 95% CI 1.06–2.76), low birth weight (OR 1.41, 95% CI 0.90–2.21), miscarriage (OR 3.5, 95% CI 1.15–10.77), stillbirth (OR 2.71, 95% CI 1.44–5.10) and neonatal death (OR 3.03, 95% CI 1.17–7.83) [55,58]. More severe maternal disease has been associated with worse fetal outcomes. Compromised vascular permeability can facilitate viral entry into the placenta while the increased production of pro-inflammatory cytokines could stimulate uterine activation proteins, leading to pre-term delivery [59,60]. Placental insufficiency can also lead indirectly to fetal hypoxia and developmental issues. Congenital neurological malformations have been described but are thought to be less common than for some other flaviviruses [61,62]. 

#### 4.3.3. Neonate

Although not well characterized, neonatal dengue fever can present with fever, thrombocytopenia, and petechiae [56,63,64]. Breastfeeding is also a potential mode of DENV transmission [65]. Infants born with maternal anti-DENV antibodies who are subsequently infected with a different DENV serotype may experience a severe disease course, akin to secondary dengue in adult populations [66,67,68].

## 5. Japanese Encephalitis Virus

### 5.1. Epidemiology

JEV is an important cause of viral encephalitis in South-East Asia and the Western Pacific Region, where it is estimated to cause approximately 70,000 clinical cases and 20,000 deaths annually [69]. In 2022, cases of Japanese encephalitis (JE) were described in South-Eastern Australia, suggesting increased distribution due to ongoing climate change and the evolving human-animal-environment interface [70,71,72]. JEV exists as five genotypes based on phylogenetic analysis of the viral envelope gene [73,74,75].

### 5.2. Pathogenesis

JEV is transmitted between birds and swine within enzootic cycles via mosquito vectors, predominantly *Culex* spp. [76,77]. The incubation period after a mosquito bite is 5–15 days. The virus replicates in local Langerhans cells and keratinocytes, and regional lymph nodes, after which it is carried by the lymphatic systemic into the thoracic duct and bloodstream [5,78]. JEV is neurotropic resulting in meningoencephalitis in approximately 1% of cases [79]. The mechanism of CNS penetration is unclear, however this is thought to involve transport via perivascular cells [80,81]. JEV can potentially cause persistent infection in the human vaginal and endometrial epithelium, as well as the trophoblast [82].

### 5.3. Clinical Features 

Asymptomatic infection or a self-limiting mild febrile illness are the most common manifestations of the disease (Table 1) [8]. In neuroinvasive disease, the reported mortality rate is 18% [83]. In survivors, up to half experience long-term neurological sequelae including seizures, impaired cognition, and motor impairment [84,85]. In endemic regions, encephalitis due to JEV primarily affects children or adolescents, as infection usually confers neutralizing immunity and seroprevalence increases with age. 

#### 5.3.1. Pregnant Women

Most women living in JEV-endemic areas have already been exposed or vaccinated by the time they reach childbearing age. Pregnant seronegative travelers to JEV-endemic areas thus bear the highest risk. There are limited data on the severity of JEV infection in the pregnant population, with one case described to date of eclamptic encephalopathy associated with JEV [86].

#### 5.3.2. Fetus

Sequelae of congenital infections have been well documented in swine and mice [82,87,88], but limited data are available in humans [89,90]. In two human case studies (encompassing eight JEV-affected pregnancies in total), maternal infections with JEV up to 22 weeks gestation were associated with miscarriage, while infections after this point did not cause apparent disease in the fetus or neonate [91,92]. JEV was isolated in the brain, liver, and placenta of one stillborn fetus [91].

#### 5.3.3. Neonate 

Postnatal JEV infection is not well described. In endemic areas with high seroprevalence to JEV, many neonates will be protected by maternally derived antibodies for the first few months of life, as is well established for transplacental transmission of other virus-specific antibodies [36,37]. A four-month-old infant was the youngest patient diagnosed with JEV encephalitis in Australia in 2022 in the context of a nascent JEV outbreak (and hence likely JEV-seronegative mother) [93]. This case highlights the vulnerability of neonates and infants to JEV when outbreaks occur in largely seronegative populations or indeed in infants traveling from non-endemic areas to regions with JEV circulation.

## 6. West Nile Virus

### 6.1. Epidemiology

First isolated in 1937 in the West Nile province of Uganda, WNV is one of the most widely distributed flaviviruses (Figure 1B). Endemic throughout Africa, the Middle East, Europe, South Asia, and Australia [94], its emergence in the Americas was first documented in 1999 after which it established endemicity [95], with seasonal peaks in summer to autumn [96]. Intermittent outbreaks involving thousands of acute infections are well documented globally [94]. Particular WNV subvariants are known to circulate in restricted geographic areas and may confer a distinct clinical phenotype, such as the Kunjin variant present in Australia which is thought to be associated with a milder disease course [97].

### 6.2. Pathogenesis

Birds have been established as a key reservoir and amplifying host of the virus [98], with transmission predominantly via mosquitoes. Direct human-to-human transmission has not been reported but transmission through infected blood and solid organ donation has been described [94].

WNV can cause neuroinvasive disease, affecting both neurons and glial cells [99]. Neuronal loss occurs via apoptosis, particularly affecting the cerebral cortex, hippocampus, cerebellum, brainstem, and spinal cord. 

### 6.3. Clinical Features

Most WNV cases are asymptomatic (80%) or manifest with a mild febrile illness associated with headache, malaise, and/or nausea (Table 1) [100]. CNS involvement, largely comprising meningoencephalitis or acute flaccid paralysis, can take a severe or sometimes fulminant course [100]. The population mortality rate from WNV is reported as 5%, rising to 9% in those with neuroinvasive disease [96]. WNV-associated flaccid paralysis in particular carries high mortality and long-term morbidity [96]. 

#### 6.3.1. Pregnant Women

In pregnancy, disease manifestations of WNV appear no more severe than in the general population. Cases of meningoencephalitis have been described [101,102].

#### 6.3.2. Fetus

Vertical transmission of WNV with associated subsequent congenital anomalies is well documented although it occurs less commonly than for other flaviviruses such as ZIKV [103]. In one case series of 72 neonates born to mothers with maternal WNV infections, three (4%) were infected with WNV with associated congenital defects: lissencephaly and encephalitis (one case), coarctation of the aorta (one case) and postnatal meningitis (one case) [104]. One further case report of congenital WNV infection involved cerebral atrophy and chorioretinitis [105]. 

#### 6.3.3. Neonate

In pediatric populations generally, the spectrum of disease due to WNV is similar to that seen in adults, although neuroinvasive disease is more likely to manifest as meningitis than encephalitis [106]. Postnatally acquired WNV infection in neonates is poorly described, with a single case report of transmission of WNV from breast milk with the infant remaining well [107].

## 7. Yellow Fever Virus

### 7.1. Epidemiology

YFV is endemic to over forty countries in tropical and subtropical Africa and South America (Figure 1A). Current modeling suggests an incidence of 200,000 cases annually with sporadic epidemics occurring most recently in Angola, the Democratic Republic of Congo and Brazil [108,109,110,111].

### 7.2. Pathogenesis

The natural reservoir for YFV are human and non-human primates, and there are two main transmission cycles: the sylvatic cycle and the urban cycle [112]. Transmission occurs via the bite of an infected mosquito (typically *Aedes* spp.) [113]. YFV is transported to regional lymph nodes where multiplication occurs followed by a primary viremia. The virus then spreads to the visceral organs where further replication occurs, with a particular tropism for hepatocytes [114]. Subsequent cytokine release causes apoptosis of the hepatocytes together with activation and consumption of clotting factors [114,115]. YFV is classified as a viral hemorrhagic fever due to the severe clinical manifestations associated with these latter stages.

### 7.3. Clinical Features

The incubation period for YFV is 3–6 days (Table 1) [116]. Most infected individuals are asymptomatic or experience a mild self-limiting disease. Symptomatic patients initially present with nonspecific symptoms including fever, headache, lethargy, myalgia, arthralgia, and vomiting which last for approximately one week. Viremia occurs within the first three days of symptom onset and may persist in severe cases [117]. In 15–20% of patients, a brief remission of up to 48 h is followed by the onset of severe symptoms, characterized by high fevers, jaundice, coagulopathy, shock, and multiorgan failure; hemorrhagic manifestations are well described [110,115]. Mortality in this group is 20–50% [110,118]. 

#### 7.3.1. Pregnant Women

There are little data on particular risk factors for YFV infection in pregnancy. It is not clear if pregnancy confers an altered spectrum of disease or clinical outcomes for the mother compared to the non-pregnant population [119].

#### 7.3.2. Fetus

There are minimal data on fetal outcomes following YFV infection during pregnancy. There are only two case reports of vertical transmission of YFV: both cases were asymptomatic at birth, then developed severe infection with fever, multiorgan failure, and coagulopathy, and subsequently died [120,121]. 

#### 7.3.3. Neonate

Postnatal acquisition of YFV in the neonatal period is poorly characterized with no case reports published to date. For infants born in endemic areas, maternally derived antibodies may be protective, however, definitive data are awaited. Travelers to endemic areas without prior YFV exposure should be aware of the potential risk of YFV infection in neonates. 

## 8. Other Flaviviruses in Pregnancy

There is a range of other human flaviviruses that have not been covered in this review due to limited published data pertaining to their impact on pregnancy and the neonatal period. Pregnancy-specific data on St Louis encephalitis virus and Murray Valley encephalitis virus are lacking as well as other less common mosquito-borne flaviviruses such as Spondweni, Usutu, Ilheus and Rocio viruses. The similarity of this latter group of viruses to ZIKV, WNV and JEV raises concerns regarding their epidemic capability and potential for pregnancy-associated morbidity, and this warrants ongoing vigilance [1]. Similarly, data are awaited on pregnancy-specific impacts of the tick-borne flaviviruses including Tick-borne encephalitis virus, Kyasanur Forest disease virus, Alkhurma hemorrhagic fever virus, louping-ill virus, Omsk hemorrhagic fever virus, and Powassan virus. If infection with one of these flaviviruses is diagnosed during pregnancy or the neonatal period, or if a concerning contact history is elicited, expert opinion should be sought to consider the potential impact on the pregnant woman, fetus, and neonate.

## 9. Laboratory Diagnosis

Flavivirus diagnosis relies on a multimodal framework with serology forming the mainstay; molecular techniques and viral culture are additional and important diagnostic tools (Table 2). DENV antigen testing is a first-line investigation for acute dengue virus infection [122]—assessing for the presence of the non-structural protein 1 (NS1) antigen—but antigen detection does not have a routine diagnostic role for the other flaviviruses. 

### 9.1. Serology

Serological testing in pregnant women follows the same algorithms used in the broader population. Gestational age at the time of acute and convalescent sera sampling informs risk stratification for the mother, fetus, and newborn if a seroconversion is demonstrated, as previously discussed for each of the flaviviruses.

Various platforms are available for flavivirus serological testing, including enzyme-linked immunosorbent assays (ELISA), immunofluorescent techniques, hemagglutination inhibition methodologies, and neutralization assays. Flavivirus serology is highly sensitive with IgM detectable within a few days of illness onset in most cases [110,117]. However, similar antigens and hence overlapping epitopes between the flaviviruses results commonly in cross-reacting antibodies, limiting assay specificity [123]. Where vaccines have been administered, vaccine-induced IgM can persist for years following vaccination [124]. Where flavivirus infection is suspected, a detailed clinical, travel, exposure, and vaccination history must be sought to determine the possible viruses causing the patient’s clinical syndrome and to assist in the interpretation of serological results. 

A pan-flavivirus serological assay may be used as an initial screen but this must be followed by more specific assays to achieve an accurate diagnosis [125]. Virus-specific IgM and IgG titers should be obtained for each of the flaviviruses of concern and tested in parallel on serum taken in the acute and convalescent period, 2–4 weeks later. Where available, plaque reduction neutralization assays may provide additional specificity and obviate some of the concerns of cross-reactivity [126]. Antibody avidity testing is available for DENV, and dengue-specific IgG avidity can assist in differentiating primary from secondary disease. DENV-specific IgM/IgG ratios may also be used for this purpose [127,128,129]. For some flaviviruses, such as JEV and WNV, serological testing of cerebrospinal fluid (CSF) for virus-specific IgM and IgG may also be performed in parallel to testing of sera [123,130].

Interpretation of flavivirus serology is very complex requiring integration of detailed clinical information including vaccination status, comparative assessment of virus-specific antibody titers across the different flaviviruses under consideration, serial sampling with parallel testing, and a thorough understanding of the strengths and constraints of the assays in use. A single positive antibody titer alone is not diagnostic, rather can be suggestive of a particular flavivirus infection providing other potential flaviviruses have been assessed.

### 9.2. Molecular Testing

Neutralizing antibodies are rapidly produced following most flavivirus infections, resulting in a short-lived and low-level viremia [131]. A sampling of blood, CSF, and urine for NAAT in the first week of illness is recommended, as is early targeted tissue sampling in cases of the organ-specific disease, such as meningoencephalitis [125]. NAAT testing of placental tissue can be highly informative in suspected congenital infections. Viral shedding can be prolonged: for example, ZIKV RNA may be detectable for long periods in urine (median 24 days) and semen (median 25 days, but up to 370 days) [132]. Targeted NAAT assays carry higher diagnostic specificity but lower sensitivity due to short-lived viremia in many of these infections [5,125]. Loop-mediated isothermal amplification (LAMP)-based methodologies have been used for the detection of some flaviviruses such as JEV in resource-limited settings [133].

Non-targeted methods such as metagenomic sequencing carry promise in unveiling occult flavivirus infection if targeted sampling with expert guidance is performed, such as in cases of unexplained meningoencephalitis [5,134]. 

### 9.3. Other Modalities

Viral culture has a role to play in flavivirus diagnosis, though it is only performed in specialized laboratories due to technical expertise and biosafety concerns. CSF, blood, urine, and tissue are appropriate specimen types. Samples are typically incubated in mosquito cell lines such as C6/36 at 28 °C for 3–4 days [135]. Cytopathic effect is often absent in mosquito cell lines, hence NAAT or serological methods are used to assess viral growth. In tissue-invasive disease such as encephalitis or suspected congenital infections such as CZS, histopathology may also be useful with immunohistochemistry to assess for the presence of viral antigens and can provide helpful information on the cellular localization of viral moieties [110,117].

### 9.4. Integration of Diagnostic Results

For most flaviviruses, seroconversion or a four-fold rise in virus-specific IgG titers in serum is diagnostic of infection, as is the detection of virus by culture or molecular methods or (for diagnosis of DENV in particular) the presence of NS1 antigen in serum or plasma [135,136]. The presence of a flavivirus-specific IgM in serum or CSF in the absence of IgM for alternative flaviviruses is highly suggestive of flavivirus infection [135,136]. 

It is the combination of results from multiple testing modalities and across a range of flavivirus targets and timepoints that enables an accurate diagnosis of infection in most cases. For example, NS1 antigen detection is sensitive for the diagnosis of primary dengue infection, while DENV-specific IgG tends to increase slowly [137]. In secondary dengue infections, NS1 antigen is less frequently detected or detected at lower levels, while DENV-specific IgG titers increase rapidly [129,138]. DENV-specific IgM/IgG ratios and IgG avidity testing may also differentiate between primary and secondary diseases [127,128,129]. An understanding of the relative merits and constraints of each of the diagnostic modalities as well as a thorough knowledge of epidemiology related to the patient’s travel or region of residence is critical to achieving a robust diagnosis.

## 10. Treatment of Flavivirus Infections

Supportive care, comprising symptomatic treatment and intravenous fluid optimization are the principles of management. In CNS involvement, such as in Japanese encephalitis, seizure control, optimization of cerebral perfusion, and prevention of secondary complications are important in supportive management [139]. 

There are no specific antiviral therapies with demonstrated efficacy against flavivirus infections [139,140,141,142]. Novel small molecules are under investigation for YFV; however, none are yet available clinically [110,143]. Studies of corticosteroids and intravenous immunoglobulin use for neuroinvasive WNV disease have been conducted but have not shown benefit [144,145]. Monoclonal antibodies are also under investigation for the treatment of many of the flaviviruses, including DENV, ZIKV, JEV, WNV and YFV, with promising in vitro data [146,147,148,149,150].

Where neurological involvement has been demonstrated, targeted neurological rehabilitation programs may be key to optimizing functional outcomes for patients [151].

### Specific Considerations in Dengue Virus Infection

In DENV infection, where progression to shock or hemorrhagic manifestations are of concern, pregnant patients should be considered for closer inpatient monitoring with serial clinical assessments and full blood counts since early recognition of progression to severe disease is vital. While paracetamol can be used to manage fever and myalgias, aspirin or other non-steroidal anti-inflammatory drugs should be avoided in DENV due to the risk of bleeding complications. Intravenous fluids may be required in severe dengue, but excessive fluid can lead to hypervolemia as vascular permeability recovers. Blood transfusion and correction of bleeding diathesis with platelet transfusion or vitamin K may also be required in cases with active hemorrhage, especially around the time of delivery [152]. Elective delivery should be deferred if possible and there may be a role for tocolytic agents in postponing labor [153]. Intravenous oxytocin analogues can reduce the risk of postpartum hemorrhage [154].

## 11. Prevention of and Vaccination for Flavivirus Infections

### 11.1. Behavioural, Environmental, and Infection Control Strategies

Avoidance of mosquito bites through behavioral measures is critical to preventative efforts. Measures include wearing long sleeves and trousers, use of insect repellent, staying indoors when feasible, particularly at dawn and dusk, and use of mosquito nets [155,156,157,158,159]. For ZIKV-infected persons in particular, mosquito bite avoidance during the viremic period has been demonstrated to reduce further transmission to other persons particularly in the first week of illness in endemic areas (or for a three-week period after return to a non-endemic area) [136,160,161].

Environmental controls to reduce mosquito breeding sites should also be implemented, including the removal of stagnant water bodies [158]. Such measures have been demonstrated to limit the spread of viruses such as DENV at the population level [162]. For WNV, blood donor screening has been implemented in some endemic areas such as the United States [95]. While expensive, this practice has vastly reduced the risk of transmission via blood products.

Prevention of sexual transmission is important to limit the spread of ZIKV. Men should avoid unprotected sex for 3–6 months and women for 2–6 months following symptom onset of an acute ZIKV infection [136,160,161], or since last exposure for persons potentially exposed to ZIKV (through travel to an endemic area or sexual contact with an infected case). Unprotected sex with a pregnant partner should be avoided for the duration of the pregnancy if a male or female partner has been infected or exposed to ZIKV (including via travel to or residence in areas of endemicity or a contemporaneous outbreak). Blood or organ donation should be avoided for four weeks following return from an endemic area [136,160,161].

### 11.2. Vaccination for Flaviviruses

Vaccine development is an area of great potential although successful candidates have been developed only for a small number of flaviviruses to date, specifically YFV, JEV and DENV.

An effective live attenuated (17D strain) vaccine is available for YFV and elicits a durable, life-long adaptive immune response [159]. The vaccine has historically been contraindicated in pregnancy, breastfeeding mothers, and infants under six months of age, as YFV RNA has been detected in breastmilk following vaccination [163] and there have been several cases of vaccine-associated encephalitis in newborns of recently vaccinated mothers [110,164,165]. However, recent studies of pregnant women receiving YFV vaccination have demonstrated no increased risk of fetal adverse events, and thus vaccination should be considered in the setting of severe epidemics or unavoidable travel to high-risk areas given the risk of severe disease in unvaccinated populations [166,167,168,169,170,171]. 

There are several second-generation JEV vaccines in use that are safe, well-tolerated, and effective [80,172,173,174]. These include live attenuated vaccines—SA 14–14-2 (Chengdu Institute of Biological Products, Chengdu, China) [175] and JE-CV (IMOJEV^®^, Sanofi-Aventis, Paris, France) [176,177] and inactivated vaccines—IXIARO^®^ (Valneva, Saint-Herblain, France) or JESPECT^®^ (Seqirus, Maidenhead, UK) [178,179]. As live vaccines, SA 14–14-2 and JE-CV are contraindicated in pregnancy, whereas IXIARO^®^ or JESPECT^®^ can be safely used [178,179]. One retrospective review of JEV vaccination in 513 pregnant military women in the United States (using either a first-generation inactivated mouse brain-derived vaccine or IXIARO^®^) demonstrated an increased risk of low birth weight in neonates [180].

There are currently no approved ZIKV vaccines, although various candidate vaccines are under development including mRNA, DNA-based, inactivated, and viral vector vaccines [181,182]. One mRNA-based ZIKV vaccine (mRNA-1893, Moderna), has recently entered phase 2 clinical trials after demonstrating good tolerability and robust neutralizing antibody responses in phase 1 studies [181]. Similarly, high seroconversion rates (100%) were demonstrated in phase 1 trials of a two-course regimen of a viral vector vaccine (Ad26.ZIKV.001, Janssen), while DNA-based vaccines (GLS-5700, Inovio Pharmaceuticals; VRC5288, NIAID; and VRC5283, NIAID) showed moderate to high seroconversion rates (60–100%) with high antibody titers. A wider range of seroconversion responses (10–100%) was demonstrated for the Zika-purified inactivated virus (ZPIV) vaccine with lower antibody titers [182].

There has been much interest in developing WNV vaccines. Among six vaccine candidates to date, the largest body of safety and immunogenicity data have been obtained for two live attenuated chimeric candidates, ChimeriVax-WN02 (Sanofi Pasteur, Lyon, French) and WN/DEN4D30 (NIAID) [183]. None have progressed yet beyond phase 2 trials.

The development of a DENV vaccine has been difficult due to the requirement to cover all four serotypes given the severe nature of secondary infections [184]. The live attenuated tetravalent chimeric vaccine, CYD-TDV (Dengvaxia^®^, Sanofi Pasteur, Lyon, French) [185], has thus only been approved to prevent secondary infections in patients previously exposed to DENV. Vaccine recipients without previous dengue infection have been shown to experience an increased risk of dengue hospitalization or severe dengue fever compared to the unvaccinated group in trials investigating its role in primary prevention [186]. These findings, and subsequent public messaging, have led to variable outcomes across different regions, with a severe and detrimental effect on public vaccine confidence in countries such as the Philippines in 2017–2018 [187]. Preliminary data on a newer recombinant live attenuated chimeric vaccine, TAK-003 (QDENGA^®^, Takeda, Chuo, Japan), also incorporating all four DENV serotypes [188], suggests efficacy in preventing symptomatic dengue, without the same safety concerns in DENV-naïve patients [74]. Other live attenuated tetravalent DENV vaccines are in clinical development [73]. Although live vaccines are contra-indicated in pregnancy, no evidence of increased adverse pregnancy outcomes was noted after the inadvertent administration of CYD-TDV to a small number of women in early pregnancy (58 exposed pregnancies in the CYD-TDV group compared to 30 in the placebo group) [77].

Further research is urgently needed to afford safe and effective vaccines against a broader range of flaviviruses given their marked impact on the population, including pregnant women, neonates, and children. Current difficulties associated with phase 3 trial execution due to short, seasonal flavivirus outbreaks that are geographically restricted and variable from year to year [183] may be overcome through international cooperation and dedicated funding streams. Pregnant women and children must be specifically considered in future vaccine trials to ensure robust data are accumulated to guide preventative efforts in these vulnerable subpopulations. 

## 12. Arthropod Vectors Responsible for Flavivirus Transmission

Flaviviruses are zoonoses transmitted to humans by the bite of arthropods, primarily mosquitoes. The endemic regions for the different flaviviruses (Figure 1) are in large part determined by the distribution of the relevant competent vector(s) and vector viral competence, as well as endemic habitats of the reservoir host(s).

The primary mosquito vector for both ZIKV and DENV is *Aedes aegypti*, endemic to South and Central America, South, and South-East Asia, the Western Pacific, Africa, and France [15,44]. This species of mosquito is also resident in a range of other countries including Australia, East Asia, and parts of the Middle East where ZIKV has yet to establish endemicity. *Aedes albopictus,* which lives across a range of temperate climates, is not the primary vector but is capable of DENV and ZIKV transmission [16,189].

*Aedes* spp. (particularly *Ae. aegypti*) are also the typical vectors for YFV transmission [113], although outbreaks due to *Haemagogus* spp. mosquitoes (*Hg. leucocelaenus* and *Hg. janthinomys*) have been well described, particularly in Brazil [190].

A wide range of mosquito species are competent vectors for JEV, although *Culex* spp. are predominantly responsible for transmission [76,77]. The relative contribution of different species of the *Culex* genus varies by geography, and other genera may also transmit the virus, including *Aedes* spp., *Armigeres* spp., *Anopheles* spp., and *Mansonia* spp. [191].

Similarly, WNV is transmitted by a wide range of mosquito species with *Culex* spp. the most important vector [94]. *Aedes* mosquitoes are also competent vectors for WNV. While in vitro transmission via ticks has been shown, their role in natural transmission is uncertain.

The distribution of arboviral vectors is heavily influenced by climatic and epidemiological factors [189]. Climate change, both gradual and episodic events, migration patterns of reservoir species (such as migratory water birds), human population movements and changes in agricultural practices can also drive expansion and shifts in flavivirus distribution [192]. Active surveillance for emergent flavivirus outbreaks in new geographical regions is critical, particularly in the context of unseasonal rainfall and flooding, extreme weather events and the broader context of global warming.

## 13. Conclusions

Flaviviruses form a diverse group of zoonotic arboviruses with variable but potentially severe impacts in pregnancy and the neonatal period. As each of the flaviviruses has its associated tissue tropism and pathophysiological pathways, their impacts on the pregnant woman, fetus and newborn are not generalizable across this genus of viruses. Some flaviviruses, such as DENV, may cause severe disease in the pregnant woman, with significant implications for maternal and fetal health. Increased fetal loss is observed in infections due to ZIKV, DENV and WNV viruses. For ZIKV, infection may lead to severe congenital infection with long-term anatomical defects and neurodevelopmental consequences. Susceptibility of the neonate to postnatal exposure and infection must also be considered, particularly when the baby is born premature and/or to a seronegative mother. Close collaboration between infectious diseases, virology, obstetric and neonatal colleagues is necessary to diagnose and manage acute infection, and long-term pediatric follow-up may be required for affected infants.

Public health responses must incorporate preventative measures with efforts to identify effective vaccine candidates. Vaccination strategies must specifically consider pregnant women and children as two high-risk groups, and breastfeeding recommendations must also account for possible transmission to the infant.

An understanding of flavivirus epidemiology, vector characteristics, clinical syndromes, and prevention and management strategies in the pregnancy and neonatal period is critical. Furthermore, although historical patterns of endemicity are well established for most flaviviruses, their geographical reach is heavily dependent on the overlap in habitats of their vector arthropods and reservoir hosts. Hence, the current and future impacts of climate change must be considered in the expansion of potential habitats as well as the potential for sporadic outbreak events [192].

## Figures and Tables

**Table 1:** Features of flavivirus infections in pregnancy.

Virus	Zika Virus (ZIKV)	Dengue (DENV)	Japanese Encephalitis Virus (JEV)	West Nile Virus (WNV)	Yellow Fever Virus (YFV)
Incubation time	2–14 days	5–7 days	5–15 days	2–14 days	3–6 days
Maternal clinical features	Asymptomatic (majority) Fever, pruritic rash (palms and soles involved), arthralgia, headache GBS or (rarely) meningoencephalitis	Significant increase in severe disease and maternal mortality in pregnancy	Asymptomatic or mild disease (majority) Meningoencephalitis in <1%	Asymptomatic (majority) Fever, headache, malaise, nausea Meningitis, encephalitis, acute flaccid paralysis (rare)	Asymptomatic (majority) Nonspecific febrile illness Severe (15% patients)—jaundice, multi-organ failure, coagulopathy
Features of congenital and neonatal infection	Severe congenital anomalies (26–65%) including ventriculomegaly, microcephaly, intracranial calcifications, ophthalmological anomalies, and neuro-developmental abnormalities Fetal loss (7–14%)	Significant association with prematurity, low birth weight, miscarriage or foetal deathCongenital malformations and neonatal transmission described, but uncommon	Miscarriage described up to 22 weeks of pregnancy; minimal data on neonatal disease	Rare congenital abnormalities (~4%): lissencephaly, meningoencephalitis, cerebral atrophy, chorioretinitis, coarctation of the aorta	Minimal data
Primary vector(s)	Mosquito (*Aedes aegypti*)	Mosquitos, primarily *Aedes* spp.	Mosquitos, primarily *Culex* spp.	Wide range of mosquitos including *Culex* spp. and *Aedes* spp. Ticks implicated in vitro but no in vivo transmission determined	Mosquito (*Aedes* spp., *Haemogogus* spp.)
Pathophysiology (unique features)	Tropism for nervous tissue (especially neural progenitor cells) and placental cells	Severe infection with dengue hemorrhagic fever or dengue shock syndrome, more common in secondary infections	Perivascular transport across the blood-brain barrier into CNS; transplacental infection well described in animal models	Neuronal apoptosis with parenchymal inflammation predominantly affecting subcortical structures; glial cell damage	Apoptosis of mid-zone hepatocytes
Diagnostic tools	Antibody detection NAAT Viral culture	Antibody detection NS1 antigen NAAT Viral culture	Antibody detection NAAT, LAMP Viral culture	Antibody detection NAAT Viral culture	Antibody detection NAAT Viral culture
Treatment options	Supportive care	Supportive care Avoid non-steroidal anti-inflammatory medications Blood products and vitamin K may be required	Supportive care	Supportive care	Supportive care
Vaccination/prevention	Vector control/reducing mosquito exposure Avoid unprotected sex following exposure or acute infection	Vector control/reducing mosquito exposure Vaccine available, however significant concern in DENV-naïve patients due to the risk of precipitating severe secondary infection; live vaccine contraindicated in pregnancy	Vector control/reducing mosquito exposure Multiple vaccines available; IXIARO/JESPECT can be used in pregnancy	Vector control/reducing mosquito exposure	Vector control/reducing mosquito exposure Live attenuated vaccine contraindicated in pregnancy (unless high-risk/severe epidemic) and contraindicated in breastfeeding and infants <6 months

Key: CNS, central nervous system; DENV, Dengue Virus; GBS, Guillain-Barré syndrome; JEV, Japanese encephalitis virus; LAMP, loop-mediated isothermal amplification; NAAT, nucleic acid amplification test; NS1, non-structural antigen 1; WNV, West Nile virus; YFV, Yellow fever virus.

**Table 2:** Characteristics of available assays for the diagnosis of flavivirus infection.

Virus	Assay	Sample Types	Advantages	Disadvantages	Comments
**Zika virus (ZKV)**	ZKV IgM ZKV IgG	Serum	IgM detectable within the first few days of illness onset, persists for several weeks to months	Cross-reaction with other flaviviruses requires careful interpretation.	Acute and convalescent titers (at 2–4 weeks from illness onset) should be compared.
	NAAT	Urine, blood, placental tissue, fetal tissue, amniotic fluid, semen, genital tract secretions, saliva	Highly sensitive	Requires fresh tissue (not formalin-fixed).	Not currently used as a first-line assay. Reserved for acutely unwell patients, for confirmatory testing, or in the diagnosis of congenital Zika syndrome (CZS).
**Dengue virus (DENV)**	NS1 antigen	Serum	Highly sensitive in the early phase of illness (primary and secondary infections). Highly specific for DENV.	Does not differentiate between dengue serotypes.	NS1 antigenemia is shorter in secondary than in primary infection
	DENV IgM DENV IgG	Serum	IgM detectable within the first few days of primary infection, and persists for 2–3 months. Rapid rise in DENV IgG in secondary infection	Cross-reaction with other flaviviruses requires careful interpretation. Vaccine-induced IgM can persist for years.	Acute and convalescent titers (at 2–4 weeks from illness onset) should be compared. IgM/IgG ratio may be used to differentiate primary (high ratio) from secondary (low ratio) infections.
	DENV IgG avidity	Serum	Can help differentiate primary from secondary disease	Only available in specialist centres.	High avidity suggests secondary infection.
	NAAT	Blood, urine	Highly specific	Low sensitivity outside the first week of illness. Expensive, not routinely available.	Not currently used as a first-line assay. Reserved for acutely unwell patients or confirmatory testing.
**Japanese encephalitis virus (JEV)**	JEV IgM JEV IgG	Serum, CSF (IgM) Serum (IgG)	IgM detectable within the first few days of illness onset	Cross-reaction with other flaviviruses requires careful interpretation. Vaccine-induced IgM can persist for years.	Acute and convalescent titers (at 2–4 weeks from illness onset) should be compared.
	NAAT	CSF, blood, urine, brain tissue	Highly specific for JEV	Low sensitivity outside first few days of illness. Invasive sampling for cerebral tissue.	Early tissue sampling enhances diagnostic yield.
**West Nile virus (WNV)**	WNV IgM WNV IgG	Serum, CSF (IgM) Serum (IgG)	IgM detectable within the first few days of illness onset	Cross-reaction with other flaviviruses requires careful interpretation.	Acute and convalescent titers (at 2–4 weeks from illness onset) should be compared.
	NAAT	Blood, urine, CSF	Highly specific	Low sensitivity outside the first week of illness. Expensive, not routinely available.	Not currently used as a first-line assay. Reserved for acutely unwell patients or for confirmatory testing.
**Yellow fever virus (YFV)**	YFV IgM YFV IgG	Serum	IgM detectable within the first few days of illness onset	Cross-reaction with other flaviviruses requires careful interpretation. Vaccine-induced IgM can persist for years.	Acute and convalescent titers (at 2–4 weeks from illness onset) should be compared.
	NAAT	Blood, urine	Highly specific	Low sensitivity outside the first week of illness. Expensive, not routinely available.	Not currently used as a first-line assay. Reserved for acutely unwell patients or for confirmatory testing.
**All**	Pan-flavivirus IgM Pan-flavivirus IgG	Serum	Can be used as a screening assay	Does not differentiate between flaviviruses.	A positive result should be further delineated with specific IgM and IgG for each Flavivirus of interest to enable accurate diagnosis. Acute and convalescent titers (at 2–4 weeks from illness onset) should be compared.
	Viral culture	CSF, tissue, blood, urine, placental tissue (ZKV), fetal tissues (ZKV)	High specificity; provides viral isolate for further sequencing	Lower sensitivity than NAAT or serology. Requires specialist laboratory with PC3 facilities.	Staff handling viral cultures should be vaccinated when possible.
	Histopathology, immunohistochemistry	Tissue	Provides detailed structural information. Immunohistochemistry provides specificity for select viral antigens	Histopathological changes are largely non-specific between flaviviruses.	Requires specialist histopathologist expertise.
	Next-generation metagenomic sequencing	Brain tissue, CSF	High specificity Pathogen-agnostic testing	High level of technical expertise is required. High cost. Available in specialist centres only.	Not appropriate for testing samples from non-sterile sites (for example respiratory secretions) or with anticipated low viral loads (for example, serum or urine).

Key: CSF, cerebrospinal fluid; CZS, congenital Zika syndrome; DENV, Dengue virus; JEV, Japanese encephalitis virus; NAAT, nucleic acid amplification test; NS1, non-structural antigen 1; PC3, physical containment level 3; WNV, West Nile virus; YFV, Yellow fever virus.

## Data Availability

Not applicable.

## Abbreviations

CI	confidence interval
CNS	central nervous system
CSF	cerebrospinal fluid
CZS	congenital Zika syndrome
DENV	Dengue virus
DHF	dengue hemorrhagic fever
DNA	deoxyribonucleic acid
DSS	dengue shock syndrome
GBS	Guillain-Barré syndrome
JE	Japanese encephalitis
JEV	Japanese encephalitis virus
LAMP	loop-mediated isothermal amplification
NAAT	nucleic acid amplification test
NS1	non-structural antigen 1
OR	odds ratio
PC3	physical containment level 3
RNA	ribonucleic acid
WNV	West Nile virus
YFV	Yellow fever virus
ZIKV	Zika virus

## References

[1] Pierson T.C., Diamond M.S. (2020). The continued threat of emerging flaviviruses. Nat. Microbiol..

[2] Chong H.Y., Leow C.Y., Abdul Majeed A.B., Leow C.H. (2019). Flavivirus infection—A review of immunopathogenesis, immunological response, and immunodiagnosis. Virus Res..

[3] European Centre for Disease Prevention and Control (2022). West Nile Virus Infections in EU/EEA and EU-Neighbouring Countries.

[4] World Health Organization (2022). Countries and Territories with Current or Previous Zika Virus Transmission.

[5] Howard-Jones A.R., Pham D., Jeoffreys N., Eden J.-S., Hueston L., Kesson A.M., Nagendra V., Samarasekara H., Newton P., Chen S.C.A. (2022). Emerging Genotype IV Japanese Encephalitis Virus Outbreak in New South Wales, Australia. Viruses.

[6] Endy T.P., Chunsuttiwat S., Nisalak A., Libraty D.H., Green S., Rothman A.L., Vaughn D.W., Ennis F.A. (2002). Epidemiology of inapparent and symptomatic acute dengue virus infection: A prospective study of primary school children in Kamphaeng Phet, Thailand. Am. J. Epidemiol..

[7] Mostashari F., Bunning M.L., Kitsutani P.T., Singer D.A., Nash D., Cooper M.J., Katz N., Liljebjelke K.A., Biggerstaff B.J., Fine A.D. (2001). Epidemic West Nile encephalitis, New York, 1999: Results of a household-based seroepidemiological survey. Lancet.

[8] Rosen L. (1986). The natural history of Japanese encephalitis virus. Annu. Rev. Microbiol..

[9] Chan-Tack K.M., Forrest G. (2006). West Nile virus meningoencephalitis and acute flaccid paralysis after infliximab treatment. J. Rheumatol..

[10] Charlier C., Beaudoin M.C., Couderc T., Lortholary O., Lecuit M. (2017). Arboviruses and pregnancy: Maternal, fetal, and neonatal effects. Lancet Child Adolesc. Health.

[11] Pardigon N. (2017). Pathophysiological mechanisms of Flavivirus infection of the central nervous system. Transfus. Clin. Biol..

[12] Osuna-Ramos J.F., Reyes-Ruiz J.M., Del Ángel R.M. (2018). The Role of Host Cholesterol During Flavivirus Infection. Front. Cell. Infect. Microbiol..

[13] Peruzzu D., Amendola A., Venturi G., de Turris V., Marsili G., Fortuna C., Fecchi K., Gagliardi M.C. (2022). Zika Virus Exploits Lipid Rafts to Infect Host Cells. Viruses.

[14] Platt D.J., Smith A.M., Arora N., Diamond M.S., Coyne C.B., Miner J.J. (2018). Zika virus-related neurotropic flaviviruses infect human placental explants and cause fetal demise in mice. Sci. Transl. Med..

[15] Centers for Disease Control and Prevention (CDC) (2022). Zika Travel Information.

[16] Bogoch I.I., Brady O.J., Kraemer M.U.G., German M., Creatore M.I., Kulkarni M.A., Brownstein J.S., Mekaru S.R., Hay S.I., Groot E. (2016). Anticipating the international spread of Zika virus from Brazil. Lancet.

[17] Centeno-Tablante E., Medina-Rivera M., Finkelstein J.L., Herman H.S., Rayco-Solon P., Garcia-Casal M.N., Rogers L., Ghezzi-Kopel K., Zambrano Leal M.P., Andrade Velasquez J.K. (2021). Update on the Transmission of Zika Virus Through Breast Milk and Breastfeeding: A Systematic Review of the Evidence. Viruses.

[18] Venturi G., Zammarchi L., Fortuna C., Remoli M.E., Benedetti E., Fiorentini C., Trotta M., Rizzo C., Mantella A., Rezza G. (2016). An autochthonous case of Zika due to possible sexual transmission, Florence, Italy, 2014. Eurosurveillance.

[19] Musso D., Nhan T., Robin E., Roche C., Bierlaire D., Zisou K., Shan Yan A., Cao-Lormeau V.M., Broult J. (2014). Potential for Zika virus transmission through blood transfusion demonstrated during an outbreak in French Polynesia, November 2013 to February 2014. Eurosurveillance.

[20] Brasil P., Calvet G.A., Siqueira A.M., Wakimoto M., de Sequeira P.C., Nobre A., Quintana Mde S., Mendonça M.C., Lupi O., de Souza R.V. (2016). Zika Virus Outbreak in Rio de Janeiro, Brazil: Clinical Characterization, Epidemiological and Virological Aspects. PLoS Negl. Trop. Dis..

[21] Edupuganti S., Natrajan M.S., Rouphael N., Lai L., Xu Y., Feldhammer M., Hill C., Patel S.M., Johnson S.J., Bower M. (2017). Biphasic Zika Illness with Rash and Joint Pain. Open Forum Infect. Dis..

[22] Parra B., Lizarazo J., Jiménez-Arango J.A., Zea-Vera A.F., González-Manrique G., Vargas J., Angarita J.A., Zuñiga G., Lopez-Gonzalez R., Beltran C.L. (2016). Guillain-Barré Syndrome Associated with Zika Virus Infection in Colombia. N. Engl. J. Med..

[23] da Silva I.R.F., Frontera J.A., Bispo de Filippis A.M., Nascimento O. (2017). Neurologic Complications Associated with the Zika Virus in Brazilian Adults. JAMA Neurol..

[24] Flamand C., Fritzell C., Matheus S., Dueymes M., Carles G., Favre A., Enfissi A., Adde A., Demar M., Kazanji M. (2017). The proportion of asymptomatic infections and spectrum of disease among pregnant women infected by Zika virus: Systematic monitoring in French Guiana, 2016. Eurosurveillance.

[25] Brasil P., Pereira J.P., Moreira M.E., Ribeiro Nogueira R.M., Damasceno L., Wakimoto M., Rabello R.S., Valderramos S.G., Halai U.A., Salles T.S. (2016). Zika Virus Infection in Pregnant Women in Rio de Janeiro. N. Engl. J. Med..

[26] Brasil P., Vasconcelos Z., Kerin T., Gabaglia C.R., Ribeiro I.P., Bonaldo M.C., Damasceno L., Pone M.V., Pone S., Zin A. (2020). Zika virus vertical transmission in children with confirmed antenatal exposure. Nat. Commun..

[27] Pomar L., Vouga M., Lambert V., Pomar C., Hcini N., Jolivet A., Benoist G., Rousset D., Matheus S., Malinger G. (2018). Maternal-fetal transmission and adverse perinatal outcomes in pregnant women infected with Zika virus: Prospective cohort study in French Guiana. BMJ.

[28] Alvarado-Domenech L.I., Rivera-Amill V., Appleton A.A., Rosario-Villafañe V., Repollet-Carrer I., Borges-Rodríguez M., Pérez-Rodríguez N.M., Olivieri-Ramos O., González M., González-Montalvo C. (2022). Early Childhood Neurodevelopmental Outcomes in Children with Prenatal Zika Virus Exposure: A Cohort Study in Puerto Rico. J. Pediatr..

[29] Ventura C.V., Ventura L.O. (2018). Ophthalmologic Manifestations Associated with Zika Virus Infection. Pediatrics.

[30] Vouga M., Baud D. (2016). Imaging of congenital Zika virus infection: The route to identification of prognostic factors. Prenat. Diagn..

[31] Freitas D.A., Souza-Santos R., Carvalho L.M.A., Barros W.B., Neves L.M., Brasil P., Wakimoto M.D. (2020). Congenital Zika syndrome: A systematic review. PLoS ONE.

[32] Hcini N., Kugbe Y., Rafalimanana Z.H.L., Lambert V., Mathieu M., Carles G., Baud D., Panchaud A., Pomar L. (2021). Association between confirmed congenital Zika infection at birth and outcomes up to 3 years of life. Nat. Commun..

[33] Pimentel R., Khosla S., Rondon J., Peña F., Sullivan G., Perez M., Mehta S.D., Brito M.O. (2021). Birth Defects and Long-Term Neurodevelopmental Abnormalities in Infants Born During the Zika Virus Epidemic in the Dominican Republic. Ann. Glob. Health.

[34] Palasanthiran P., Starr M., Jones C., Giles M., Australasian Society for Infectious Diseases (2022). Management of Perinatal Infections.

[35] Maness N.J., Schouest B., Singapuri A., Dennis M., Gilbert M.H., Bohm R.P., Schiro F., Aye P.P., Baker K., Van Rompay K.K.A. (2019). Postnatal Zika virus infection of nonhuman primate infants born to mothers infected with homologous Brazilian Zika virus. Sci. Rep..

[36] Healy C.M., Rench M.A., Swaim L.S., Timmins A., Vyas A., Sangi-Haghpeykar H., Ng N., Rajam G., Havers F., Schiffer J. (2020). Kinetics of maternal pertussis-specific antibodies in infants of mothers vaccinated with tetanus, diphtheria and acellular pertussis (Tdap) during pregnancy. Vaccine.

[37] Nyiro J.U., Sande C., Mutunga M., Kiyuka P.K., Munywoki P.K., Scott J.A., Nokes D.J. (2015). Quantifying maternally derived respiratory syncytial virus specific neutralising antibodies in a birth cohort from coastal Kenya. Vaccine.

[38] Bentes A.A., Crispim A.P.C., Marinho P.E.S., Viegas E.C.C., Loutfi K.S., Guedes I., Araujo S.T., Alvarenga A.M., Campos E.S.L.M., Santos M.A. (2021). Neurologic Manifestations of Noncongenital Zika Virus in Children. J. Pediatr..

[39] Shepard D.S., Undurraga E.A., Halasa Y.A., Stanaway J.D. (2016). The global economic burden of dengue: A systematic analysis. Lancet Infect. Dis..

[40] Hales S., de Wet N., Maindonald J., Woodward A. (2002). Potential effect of population and climate changes on global distribution of dengue fever: An empirical model. Lancet.

[41] Guzman M.G., Harris E. (2015). Dengue. Lancet.

[42] Henchal E.A., Putnak J.R. (1990). The dengue viruses. Clin. Microbiol. Rev..

[43] Simmons C.P., Farrar J.J., van Vinh Chau N., Wills B. (2012). Dengue. N. Engl. J. Med..

[44] Kuno G. (1995). Review of the Factors Modulating Dengue Transmission. Epidemiol. Rev..

[45] Vaughn D.W., Green S., Kalayanarooj S., Innis B.L., Nimmannitya S., Suntayakorn S., Rothman A.L., Ennis F.A., Nisalak A. (1997). Dengue in the Early Febrile Phase: Viremia and Antibody Responses. J. Infect. Dis..

[46] Halstead S.B. (2014). Dengue Antibody-Dependent Enhancement: Knowns and Unknowns. Microbiol. Spectr..

[47] Katzelnick L.C., Gresh L., Halloran M.E., Mercado J.C., Kuan G., Gordon A., Balmaseda A., Harris E. (2017). Antibody-dependent enhancement of severe dengue disease in humans. Science.

[48] Libraty D.H., Endy T.P., Houng H., Shu H., Green S., Kalayanarooj S., Suntayakorn S., Chansiriwongs W., Vaughn D.W., Nisalak A. (2002). Differing Influences of Virus Burden and Immune Activation on Disease Severity in Secondary Dengue-3 Virus Infections. J. Infect. Dis..

[49] Rothman A.L. (2011). Immunity to dengue virus: A tale of original antigenic sin and tropical cytokine storms. Nat. Rev. Immunol..

[50] Endy T.P., Srikiatkhachorn A., Jarman R.G., Mammen M.P., Vaughn D.W., Kalanarooj S., Nisalak A., Gibbons R.V. (2007). Analysis of Repeat Hospital Admissions for Dengue to Estimate the Frequency of Third or Fourth Dengue Infections Resulting in Admissions and Dengue Hemorrhagic Fever, and Serotype Sequences. Am. J. Trop. Med. Hyg..

[51] Martina B.E.E., Koraka P., Osterhaus A.D.M.E. (2009). Dengue Virus Pathogenesis: An Integrated View. Clin. Microbiol. Rev..

[52] Beatty P.R., Puerta-Guardo H., Killingbeck S.S., Glasner D.R., Hopkins K., Harris E. (2015). Dengue virus NS1 triggers endothelial permeability and vascular leak that is prevented by NS1 vaccination. Sci. Transl. Med..

[53] Nakao S., Lai C.J., Young N.S. (1989). Dengue virus, a flavivirus, propagates in human bone marrow progenitors and hematopoietic cell lines. Blood.

[54] Machado C.R., Machado E.S., Rohloff R.D., Azevedo M., Campos D.P., de Oliveira R.B., Brasil P. (2013). Is Pregnancy Associated with Severe Dengue? A Review of Data from the Rio de Janeiro Surveillance Information System. PLoS Negl. Trop. Dis..

[55] Rathore S.S., Oberoi S., Hilliard J., Raja R., Ahmed N.K., Vishwakarma Y., Iqbal K., Kumari C., Velasquez-Botero F., Nieto-Salazar M.A. (2022). Maternal and foetal-neonatal outcomes of dengue virus infection during pregnancy. Trop. Med. Int. Health.

[56] Basurko C., Everhard S., Matheus S., Restrepo M., Hildéral H., Lambert V., Boukhari R., Duvernois J.-P., Favre A., Valmy L. (2018). A prospective matched study on symptomatic dengue in pregnancy. PLoS ONE.

[57] Brar R., Sikka P., Suri V., Singh M.P., Suri V., Mohindra R., Biswal M. (2021). Maternal and fetal outcomes of dengue fever in pregnancy: A large prospective and descriptive observational study. Arch. Gynecol. Obstet..

[58] Paixão E.S., Teixeira M.G., Costa M.d.C.N., Rodrigues L.C. (2016). Dengue during pregnancy and adverse fetal outcomes: A systematic review and meta-analysis. Lancet Infect. Dis..

[59] Nunes P., Nogueira R., Coelho J., Rodrigues F., Salomão N., José C., de Carvalho J., Rabelo K., de Azeredo E., Basílio-de-Oliveira R. (2019). A Stillborn Multiple Organs’ Investigation from a Maternal DENV-4 Infection: Histopathological and Inflammatory Mediators Characterization. Viruses.

[60] Ribeiro C.F., Lopes V.G.S., Brasil P., Pires A.R.C., Rohloff R., Nogueira R.M.R. (2017). Dengue infection in pregnancy and its impact on the placenta. Int. J. Infect. Dis..

[61] Paixão E.S., Costa M.d.C.N., Teixeira M.G., Harron K., de Almeida M.F., Barreto M.L., Rodrigues L.C. (2017). Symptomatic dengue infection during pregnancy and the risk of stillbirth in Brazil, 2006–2012: A matched case-control study. Lancet Infect. Dis..

[62] Sharma J.B., Gulati N. (1992). Potential relationship between dengue fever and neural tube defects in a Northern District of India. Int. J. Gynecol. Obstet..

[63] Ribeiro C.F., Lopes V.G.S., Brasil P., Coelho J., Muniz A.G., Nogueira R.M.R. (2013). Perinatal Transmission of Dengue: A Report of 7 Cases. J. Pediatr..

[64] Tan P.C., Rajasingam G., Devi S., Omar S.Z. (2008). Dengue infection in pregnancy: Prevalence, vertical transmission, and pregnancy outcome. Obstet. Gynecol..

[65] Arragain L., Dupont-Rouzeyrol M., O’Connor O., Sigur N., Grangeon J.-P., Huguon E., Dechanet C., Cazorla C., Gourinat A.-C., Descloux E. (2016). Vertical Transmission of Dengue Virus in the Peripartum Period and Viral Kinetics in Newborns and Breast Milk: New Data. J. Pediatr. Infect. Dis. Soc..

[66] Jain A., Chaturvedi U.C. (2010). Dengue in infants: An overview. FEMS Immunol. Med. Microbiol..

[67] Nimmanitya S., Kliks S.C., Burke D.S., Nisalak A. (1988). Evidence That Maternal Dengue Antibodies Are Important in the Development of Dengue Hemorrhagic Fever in Infants. Am. J. Trop. Med. Hyg..

[68] Simmons C.P., Chau T.N.B., Thuy T.T., Tuan N.M., Hoang D.M., Thien N.T., Lien L.B., Quy N.T., Hieu N.T., Hien T.T. (2007). Maternal Antibody and Viral Factors in the Pathogenesis of Dengue Virus in Infants. J. Infect. Dis..

[69] Tsarev S.A., Sanders M.L., Vaughn D.W., Innis B.L. (2000). Phylogenetic analysis suggests only one serotype of Japanese encephalitis virus. Vaccine.

[70] Auerswald H., Maquart P.-O., Chevalier V., Boyer S. (2021). Mosquito Vector Competence for Japanese Encephalitis Virus. Viruses.

[71] Solomon T., Ni H., Beasley D.W.C., Ekkelenkamp M., Cardosa M.J., Barrett A.D.T. (2003). Origin and Evolution of Japanese Encephalitis Virus in Southeast Asia. J. Virol..

[72] van den Hurk A.F., Skinner E., Ritchie S.A., Mackenzie J.S. (2022). The Emergence of Japanese Encephalitis Virus in Australia in 2022: Existing Knowledge of Mosquito Vectors. Viruses.

[73] Kallas E.G., Precioso A.R., Palacios R., Thomé B., Braga P.E., Vanni T., Campos L.M.A., Ferrari L., Mondini G., da Graça Salomão M. (2020). Safety and immunogenicity of the tetravalent, live-attenuated dengue vaccine Butantan-DV in adults in Brazil: A two-step, double-blind, randomised placebo-controlled phase 2 trial. Lancet Infect. Dis..

[74] Rivera L., Biswal S., Sáez-Llorens X., Reynales H., López-Medina E., Borja-Tabora C., Bravo L., Sirivichayakul C., Kosalaraksa P., Martinez Vargas L. (2022). Three-year Efficacy and Safety of Takeda’s Dengue Vaccine Candidate (TAK-003). Clin. Infect. Dis..

[75] Whitehead S.S. (2016). Development of TV003/TV005, a single dose, highly immunogenic live attenuated dengue vaccine; what makes this vaccine different from the Sanofi-Pasteur CYD™ vaccine?. Expert Rev. Vaccines.

[76] Gould E.A., Solomon T. (2008). Pathogenic flaviviruses. Lancet.

[77] Skipetrova A., Wartel T.A., Gailhardou S. (2018). Dengue vaccination during pregnancy–An overview of clinical trials data. Vaccine.

[78] Campbell G., Hills S., Fischer M., Jacobson J., Hoke C., Hombach J., Marfin A., Solomon T., Tsai T., Tsui V. (2011). Estimated global incidence of Japanese encephalitis. Bull. World Health Organ..

[79] Mackenzie J.S., Williams D.T., van den Hurk A.F., Smith D.W., Currie B.J. (2022). Japanese Encephalitis Virus: The Emergence of Genotype IV in Australia and Its Potential Endemicity. Viruses.

[80] Kumar A., Sharma P., Shukla K.K., Misra S., Nyati K.K. (2019). Japanese encephalitis virus: Associated immune response and recent progress in vaccine development. Microb. Pathog..

[81] Le Flohic G., Porphyre V., Barbazan P., Gonzalez J.-P. (2013). Review of Climate, Landscape, and Viral Genetics as Drivers of the Japanese Encephalitis Virus Ecology. PLoS Negl. Trop. Dis..

[82] Burns K.F. (1950). Congenital Japanese B Encephalitis Infection of Swine. Exp. Biol. Med..

[83] Turtle L., Solomon T. (2018). Japanese encephalitis—The prospects for new treatments. Nat. Rev. Neurol..

[84] Sharma K.B., Vrati S., Kalia M. (2021). Pathobiology of Japanese encephalitis virus infection. Mol. Asp. Med..

[85] Solomon T. (2004). Flavivirus Encephalitis. N. Engl. J. Med..

[86] Solomon T., Winter P.M. (2004). Neurovirulence and host factors in flavivirus encephalitis—Evidence from clinical epidemiology. Emergence and Control of Zoonotic Viral Encephalitides.

[87] Mathur A., Arora K.L., Chaturvedi U.C. (1981). Congenital infection of mice with Japanese encephalitis virus. Infect. Immun..

[88] Mathur A., Arora K.L., Chaturvedi U.C. (1982). Transplacental Japanese Encephalitis Virus (JEV) Infection in Mice During Consecutive Pregnancies. J. Gen. Virol..

[89] Turtle L., Easton A., Defres S., Ellul M., Bovill B., Hoyle J., Jung A., Lewthwaite P., Solomon T. (2019). ‘More than devastating’—Patient experiences and neurological sequelae of Japanese encephalitis. J. Travel Med..

[90] Verma R., Junewar V., Praharaj H.N. (2012). Unusual association of eclamptic encephalopathy and Japanese encephalitis. Case Rep..

[91] Chaturvedi U.C., Mathur A., Chandra A., Das S.K., Tandon H.O., Singh U.K. (1980). Transplacental Infection with Japanese Encephalitis Virus. J. Infect. Dis..

[92] Mathur A., Tandon H.O., Mathur K.R., Sarkari N.B., Singh U.K., Chaturvedi U.C. (1985). Japanese encephalitis virus infection during pregnancy. Indian J. Med. Res..

[93] Zhu A., Petrakis N., Gaber M., Mason D., Clifford V., Kelly J. (2022). A case of Japanese encephalitis in a Victorian infant. Med. J. Aust..

[94] Chancey C., Grinev A., Volkova E., Rios M. (2015). The global ecology and epidemiology of West Nile virus. BioMed Res. Int..

[95] Petersen L.R. (2019). Epidemiology of West Nile Virus in the United States: Implications for Arbovirology and Public Health. J. Med. Entomol..

[96] Centers for Disease Control and Prevention (CDC) (2021). West Nile Virus: Final Cumulative Maps and Data for 1999–2020.

[97] Gray T.J., Burrow J.N., Markey P.G., Whelan P.I., Jackson J., Smith D.W., Currie B.J. (2011). West nile virus (Kunjin subtype) disease in the northern territory of Australia--a case of encephalitis and review of all reported cases. Am. J. Trop. Med. Hyg..

[98] Komar N., Langevin S., Hinten S., Nemeth N., Edwards E., Hettler D., Davis B., Bowen R., Bunning M. (2003). Experimental infection of North American birds with the New York 1999 strain of West Nile virus. Emerg. Infect. Dis..

[99] Ludlow M., Kortekaas J., Herden C., Hoffmann B., Tappe D., Trebst C., Griffin D.E., Brindle H.E., Solomon T., Brown A.S. (2016). Neurotropic virus infections as the cause of immediate and delayed neuropathology. Acta Neuropathol..

[100] Sejvar J.J. (2007). The long-term outcomes of human West Nile virus infection. Clin. Infect. Dis..

[101] Bruno J., Rabito F.J., Dildy G.A. (2004). West nile virus meningoencephalitis during pregnancy. J. La. State Med. Soc..

[102] Stewart R.D., Bryant S.N., Sheffield J.S. (2013). West nile virus infection in pregnancy. Case Rep. Infect. Dis..

[103] Jamieson D.J., Jernigan D.B., Ellis J.E., Treadwell T.A. (2005). Emerging infections and pregnancy: West Nile virus, monkeypox, severe acute respiratory syndrome, and bioterrorism. Clin. Perinatol..

[104] O’Leary D.R., Kuhn S., Kniss K.L., Hinckley A.F., Rasmussen S.A., Pape W.J., Kightlinger L.K., Beecham B.D., Miller T.K., Neitzel D.F. (2006). Birth outcomes following West Nile Virus infection of pregnant women in the United States: 2003–2004. Pediatrics.

[105] Alpert S.G., Fergerson J., Noël L.P. (2003). Intrauterine West Nile virus: Ocular and systemic findings. Am. J. Ophthalmol..

[106] Lindsey N.P., Hayes E.B., Staples J.E., Fischer M. (2009). West Nile virus disease in children, United States, 1999–2007. Pediatrics.

[107] Centers for Disease Control and Prevention (CDC) (2002). Possible West Nile virus transmission to an infant through breast-feeding--Michigan, 2002. Morb. Mortal. Wkly. Rep..

[108] de Oliveira Figueiredo P., Stoffella-Dutra A.G., Barbosa Costa G., Silva de Oliveira J., Dourado Amaral C., Duarte Santos J., Soares Rocha K.L., Araújo Júnior J.P., Lacerda Nogueira M., Zazá Borges M.A. (2020). Re-Emergence of Yellow Fever in Brazil during 2016–2019: Challenges, Lessons Learned, and Perspectives. Viruses.

[109] Douam F., Ploss A. (2018). Yellow Fever Virus: Knowledge Gaps Impeding the Fight Against an Old Foe. Trends Microbiol..

[110] Monath T.P., Vasconcelos P.F. (2015). Yellow fever. J. Clin. Virol..

[111] World Health Organization (2013). Vaccines and vaccination against Yellow fever. WHO position paper–June 2013. Wkly. Epidemiol. Rec..

[112] Gardner C.L., Ryman K.D. (2010). Yellow fever: A reemerging threat. Clin. Lab. Med..

[113] Staples J.E., Monath T.P. (2008). Yellow fever: 100 years of discovery. JAMA.

[114] Quaresma J.A., Pagliari C., Medeiros D.B., Duarte M.I., Vasconcelos P.F. (2013). Immunity and immune response, pathology and pathologic changes: Progress and challenges in the immunopathology of Yellow fever. Rev. Med. Virol..

[115] Lindenbach B.D., Murray C.L., Thiel H.J., Rice C.M., Knipe D.M., Howley P.M. (2013). Flaviviridae. Fields Virology.

[116] Rudolph K.E., Lessler J., Moloney R.M., Kmush B., Cummings D.A. (2014). Incubation periods of mosquito-borne viral infections: A systematic review. Am. J. Trop. Med. Hyg..

[117] Domingo C., Charrel R.N., Schmidt-Chanasit J., Zeller H., Reusken C. (2018). Yellow fever in the diagnostics laboratory. Emerg. Microbes Infect..

[118] Tuboi S.H., Costa Z.G., da Costa Vasconcelos P.F., Hatch D. (2007). Clinical and epidemiological characteristics of Yellow fever in Brazil: Analysis of reported cases 1998–2002. Trans. R. Soc. Trop. Med. Hyg..

[119] Krubiner C.B., Schwartz D.A. (2019). Viral Hemorrhagic Fevers in Pregnant Women and the Vaccine Landscape: Comparisons between Yellow Fever, Ebola, and Lassa Fever. Curr. Trop. Med. Rep..

[120] Bentlin M.R., de Barros Almeida R.A., Coelho K.I., Ribeiro A.F., Siciliano M.M., Suzuki A., Fortaleza C.M. (2011). Perinatal transmission of Yellow fever, Brazil, 2009. Emerg. Infect. Dis..

[121] Diniz L.M.O., Romanelli R.M.C., de Carvalho A.L., Teixeira D.C., de Carvalho L.F.A., Ferreira Cury V., Filho M.P.L., Perígolo G., Heringer T.P. (2019). Perinatal Yellow Fever: A Case Report. Pediatr. Infect. Dis. J..

[122] Hunsperger E.A., Muñoz-Jordán J., Beltran M., Colón C., Carrión J., Vazquez J., Acosta L.N., Medina-Izquierdo J.F., Horiuchi K., Biggerstaff B.J. (2016). Performance of Dengue Diagnostic Tests in a Single-Specimen Diagnostic Algorithm. J. Infect. Dis..

[123] Communicable Diseases Network Australia (CDNA) (2010). Australian National Notifiable Diseases Case Definition: West Nile/Kunjin Virus Infection.

[124] Gibney K.B., Edupuganti S., Panella A.J., Kosoy O.I., Delorey M.J., Lanciotti R.S., Mulligan M.J., Fischer M., Staples J.E. (2012). Detection of anti-Yellow fever virus immunoglobulin M antibodies at 3-4 years following Yellow fever vaccination. Am. J. Trop. Med. Hyg..

[125] Pham D., Howard-Jones A.R., Hueston L., Jeoffreys N., Doggett S., Rockett R.J., Eden J.-S., Sintchenko V., Chen S.C., O’Sullivan M.V. (2022). Emergence of Japanese encephalitis in Australia: A diagnostic perspective. Pathology.

[126] Houghton-Triviño N., Montaña D., Castellanos J. (2008). Dengue-Yellow fever sera cross-reactivity; challenges for diagnosis. Rev. Salud Publica.

[127] Changal K.H., Raina A.H., Raina A., Raina M., Bashir R., Latief M., Mir T., Changal Q.H. (2016). Differentiating secondary from primary dengue using IgG to IgM ratio in early dengue: An observational hospital based clinico-serological study from North India. BMC Infect. Dis..

[128] Matheus S.V., Deparis X., Labeau B., Lelarge J., Morvan J., Dussart P. (2005). Discrimination between Primary and Secondary Dengue Virus Infection by an Immunoglobulin G Avidity Test Using a Single Acute-Phase Serum Sample. J. Clin. Microbiol..

[129] Nguyen T.H.T., Clapham H.E., Phung K.L., Nguyen T.K., Dinh T.T., Nguyen T.H.Q., Tran V.N., Whitehead S., Simmons C., Wolbers M. (2018). Methods to discriminate primary from secondary dengue during acute symptomatic infection. BMC Infect. Dis..

[130] Centers for Disease Control and Prevention (CDC) (2021). West Nile Virus: Diagnostic Testing.

[131] Domingo C., Patel P., Yillah J., Weidmann M., Méndez J.A., Nakouné E.R., Niedrig M. (2012). Advanced Yellow fever virus genome detection in point-of-care facilities and reference laboratories. J. Clin. Microbiol..

[132] Barzon L., Percivalle E., Pacenti M., Rovida F., Zavattoni M., Del Bravo P., Cattelan A.M., Palù G., Baldanti F. (2018). Virus and Antibody Dynamics in Travelers with Acute Zika Virus Infection. Clin. Infect. Dis..

[133] Chen Z., Liao Y., Ke X., Zhou J., Chen Y., Gao L., Chen Q., Yu S. (2011). Comparison of reverse transcription loop-mediated isothermal amplification, conventional PCR and real-time PCR assays for Japanese encephalitis virus. Mol. Biol. Rep..

[134] Maamary J., Maddocks S., Barnett Y., Wong S., Rodriguez M., Hueston L., Jeoffreys N., Eden J.-S., Dwyer D.E., Floyd T. First detection of locally acquired Japanese Encephalitis Virus in New South Wales, Australia using clinical metagenomics. Emerg. Infect. Dis..

[135] Public Health Laboratory Network (2022). Flavivirus: Laboratory Case Definition.

[136] Communicable Diseases Network Australia (CDNA) (2016). CDNA National Guidelines for Public Health Units: Zika Virus Infection.

[137] Tricou V., Minh N.N., Farrar J., Tran H.T., Simmons C.P. (2011). Kinetics of Viremia and NS1 Antigenemia Are Shaped by Immune Status and Virus Serotype in Adults with Dengue. PLoS Negl. Trop. Dis..

[138] Guzman M.G., Jaenisch T., Gaczkowski R., Ty Hang V.T., Sekaran S.D., Kroeger A., Vazquez S., Ruiz D., Martinez E., Mercado J.C. (2010). Multi-Country Evaluation of the Sensitivity and Specificity of Two Commercially-Available NS1 ELISA Assays for Dengue Diagnosis. PloS Negl. Trop. Dis..

[139] Tiroumourougane S.V. (2002). Japanese viral encephalitis. Postgrad. Med. J..

[140] Chowers M.Y., Lang R., Nassar F., Ben-David D., Giladi M., Rubinshtein E., Itzhaki A., Mishal J., Siegman-Igra Y., Kitzes R. (2001). Clinical characteristics of the West Nile fever outbreak, Israel, 2000. Emerg. Infect. Dis..

[141] Popescu C.P., Florescu S.A., Hasbun R., Harxhi A., Evendar R., Kahraman H., Neuberger A., Codreanu D., Zaharia M.F., Tosun S. (2020). Prediction of unfavorable outcomes in West Nile virus neuroinvasive infection-Result of a multinational ID-IRI study. J. Clin. Virol..

[142] World Health Organization (2009). Dengue: Guidelines for Diagnosis, Treatment, Prevention and Control.

[143] Monath T.P. (2008). Treatment of Yellow fever. Antivir. Res..

[144] Gnann J.W., Agrawal A., Hart J., Buitrago M., Carson P., Hanfelt-Goade D., Tyler K., Spotkov J., Freifeld A., Moore T. (2019). Lack of Efficacy of High-Titered Immunoglobulin in Patients with West Nile Virus Central Nervous System Disease. Emerg. Infect. Dis..

[145] Murray K.O., Baraniuk S., Resnick M., Arafat R., Kilborn C., Shallenberger R., York T.L., Martinez D., Malkoff M., Elgawley N. (2008). Clinical investigation of hospitalized human cases of West Nile virus infection in Houston, Texas, 2002–2004. Vector Borne Zoonotic Dis..

[146] Doyle M.P., Genualdi J.R., Bailey A.L., Kose N., Gainza C., Rodriguez J., Reeder K.M., Nelson C.A., Jethva P.N., Sutton R.E. (2022). Isolation of a Potently Neutralizing and Protective Human Monoclonal Antibody Targeting Yellow Fever Virus. mBio.

[147] Kai Y., Lilan X., Yaoming L. (2021). Monoclonal Antibody That Inhibits Cleavage Activity of Japanese Encephalitis Virus NS3. Monoclon. Antibodies Immunodiagn. Immunother..

[148] Lu J., Chen L., Du P., Guo J., Wang X., Jiang Y., Yu Y., Wang R., Yang Z. (2022). A human monoclonal antibody to neutralize all four serotypes of dengue virus derived from patients at the convalescent phase of infection. Virology.

[149] Ozawa T., Masaki H., Takasaki T., Aoyama I., Yumisashi T., Yamanaka A., Konishi E., Ohnuki Y., Muraguchi A., Kishi H. (2018). Human monoclonal antibodies against West Nile virus from Japanese encephalitis-vaccinated volunteers. Antivir. Res..

[150] Sootichote R., Puangmanee W., Benjathummarak S., Kowaboot S., Yamanaka A., Boonnak K., Ampawong S., Chatchen S., Ramasoota P., Pitaksajjakul P. (2023). Potential Protective Effect of Dengue NS1 Human Monoclonal Antibodies against Dengue and Zika Virus Infections. Biomedicines.

[151] Christie S., Chan V., Mollayeva T., Colantonio A. (2018). Systematic review of rehabilitation intervention outcomes of adult and paediatric patients with infectious encephalitis. BMJ Open.

[152] Rajapakse S., de Silva N.L., Weeratunga P., Rodrigo C., Fernando S.D. (2017). Prophylactic and therapeutic interventions for bleeding in dengue: A systematic review. Trans. R. Soc. Trop. Med. Hyg..

[153] Escobar M.F., Mora B.L., Cedano J.A., Loaiza S., Rosso F. (2020). Comprehensive treatment in severe dengue during preterm and term labor: Could tocolysis be useful?. J. Matern.-Fetal Neonatal Med..

[154] Oladapo O.T., Okusanya B.O., Abalos E., Gallos I.D., Papadopoulou A. (2020). Intravenous versus intramuscular prophylactic oxytocin for reducing blood loss in the third stage of labour. Cochrane Database Syst. Rev..

[155] Gabiane G., Yen P.S., Failloux A.B. (2022). Aedes mosquitoes in the emerging threat of urban Yellow fever transmission. Rev. Med. Virol..

[156] Gujral I.B., Zielinski-Gutierrez E.C., LeBailly A., Nasci R. (2007). Behavioral risks for West Nile virus disease, northern Colorado, 2003. Emerg. Infect. Dis..

[157] Han L.L., Popovici F., Alexander J.P., Laurentia V., Tengelsen L.A., Cernescu C., Gary H.E., Ion-Nedelcu N., Campbell G.L., Tsai T.F. (1999). Risk factors for West Nile virus infection and meningoencephalitis, Romania, 1996. J. Infect. Dis..

[158] Kabilan L. (2004). Control of Japanese encephalitis in India: A reality. Indian J. Pediatr..

[159] Monath T.P. (2005). Yellow fever vaccine. Expert Rev. Vaccines.

[160] Petersen E.E., Meaney-Delman D., Neblett-Fanfair R., Havers F., Oduyebo T., Hills S.L., Rabe I.B., Lambert A., Abercrombie J., Martin S.W. (2016). Update: Interim Guidance for Preconception Counseling and Prevention of Sexual Transmission of Zika Virus for Persons with Possible Zika Virus Exposure-United States, September 2016. Morb. Mortal. Wkly. Rep..

[161] World Health Organization (2016). Prevention of Potential Sexual Transmission of Zika Virus: Interim Guidance.

[162] Heydari N., Larsen D.A., Neira M., Beltrán Ayala E., Fernandez P., Adrian J., Rochford R., Stewart-Ibarra A.M. (2017). Household Dengue Prevention Interventions, Expenditures, and Barriers to Aedes aegypti Control in Machala, Ecuador. Int. J. Environ. Res. Public Health.

[163] Hassan T., Bashir R., Abdelrahman D., Madni H., M El Hussein A., Elkidir I., Enan K. (2022). Transmission of Yellow fever vaccine virus from breast feeding mothers to their infants: Reporting of Yellow fever virus (YFV) RNA detection in milk specimens. F1000Research.

[164] Kuhn S., Twele-Montecinos L., MacDonald J., Webster P., Law B. (2011). Case report: Probable transmission of vaccine strain of Yellow fever virus to an infant via breast milk. Can. Med. Assoc. J..

[165] Traiber C., Coelho-Amaral P., Ritter V.R., Winge A. (2011). Infant meningoencephalitis caused by Yellow fever vaccine virus transmitted via breastmilk. J. Pediatr..

[166] Cavalcanti D.P., Salomão M.A., Lopez-Camelo J., Pessoto M.A. (2007). Early exposure to Yellow fever vaccine during pregnancy. Trop. Med. Int. Health.

[167] D’Acremont V., Tremblay S., Genton B. (2008). Impact of vaccines given during pregnancy on the offspring of women consulting a travel clinic: A longitudinal study. J. Travel Med..

[168] Nasidi A., Monath T.P., Vandenberg J., Tomori O., Calisher C.H., Hurtgen X., Munube G.R., Sorungbe A.O., Okafor G.C., Wali S. (1993). Yellow fever vaccination and pregnancy: A four-year prospective study. Trans. R. Soc. Trop. Med. Hyg..

[169] Robert E., Vial T., Schaefer C., Arnon J., Reuvers M. (1999). Exposure to Yellow fever vaccine in early pregnancy. Vaccine.

[170] Staples J.E., Gershman M., Fischer M. (2010). Yellow fever vaccine: Recommendations of the Advisory Committee on Immunization Practices (ACIP). MMWR Recomm. Rep..

[171] Suzano C.E., Amaral E., Sato H.K., Papaiordanou P.M. (2006). The effects of Yellow fever immunization (17DD) inadvertently used in early pregnancy during a mass campaign in Brazil. Vaccine.

[172] Heffelfinger J.D., Li X., Batmunkh N., Grabovac V., Diorditsa S., Liyanage J.B., Pattamadilok S., Bahl S., Vannice K.S., Hyde T.B. (2017). Japanese Encephalitis Surveillance and Immunization—Asia and Western Pacific Regions, 2016. Morb. Mortal. Wkly. Rep..

[173] Li X., Ma S.-J., Liu X., Jiang L.-N., Zhou J.-H., Xiong Y.-Q., Ding H., Chen Q. (2014). Immunogenicity and safety of currently available Japanese encephalitis vaccines: A systematic review. Hum. Vaccines Immunother..

[174] Schiøler K.L., Samuel M., Wai K.L. (2007). Vaccines for preventing Japanese encephalitis. Cochrane Database Syst. Rev..

[175] Eckels K., Yongxin Y., Dubois D., Marchette N., Trent D., Johnson A. (1988). Japanese encephalitis virus live-attenuated vaccine, Chinese strain SA14-14-2; adaptation to primary canine kidney cell cultures and preparation of a vaccine for human use. Vaccine.

[176] Appaiahgari M.B., Vrati S. (2010). IMOJEV^®^: A Yellow fever virus-based novel Japanese encephalitis vaccine. Expert Rev. Vaccines.

[177] Appaiahgari M.B., Vrati S. (2012). Clinical development of IMOJEV^®^—A recombinant Japanese encephalitis chimeric vaccine (JE-CV). Expert Opin. Biol. Ther..

[178] Erra E.O., Kantele A. (2015). The Vero cell-derived, inactivated, SA 14-14-2 strain-based vaccine (Ixiaro) for prevention of Japanese encephalitis. Expert Rev. Vaccines.

[179] Jelinek T. (2013). IXIARO^®^ updated: Overview of clinical trials and developments with the inactivated vaccine against Japanese encephalitis. Expert Rev. Vaccines.

[180] Khodr Z.G., Hall C., Chang R.N., Bukowinski A.T., Gumbs G.R., Conlin A.M.S. (2020). Japanese encephalitis vaccination in pregnancy among U.S. active duty military women. Vaccine.

[181] Essink B., Chu L., Seger W., Barranco E., Le Cam N., Bennett H., Faughnan V., Pajon R., Paila Y.D., Bollman B. (2023). The safety and immunogenicity of two Zika virus mRNA vaccine candidates in healthy flavivirus baseline seropositive and seronegative adults: The results of two randomised, placebo-controlled, dose-ranging, phase 1 clinical trials. Lancet Infect. Dis..

[182] Yeasmin M., Molla M.M.A., Masud H., Saif-Ur-Rahman K.M. (2022). Safety and immunogenicity of Zika virus vaccine: A systematic review of clinical trials. Rev. Med. Virol..

[183] Kaiser J.A., Barrett A.D.T. (2019). Twenty Years of Progress Toward West Nile Virus Vaccine Development. Viruses.

[184] McArthur M.A., Sztein M.B., Edelman R. (2013). Dengue vaccines: Recent developments, ongoing challenges and current candidates. Expert Rev. Vaccines.

[185] Thomas S.J., Yoon I.-K. (2019). A review of Dengvaxia^®^: Development to deployment. Hum. Vaccines Immunother..

[186] Sridhar S., Luedtke A., Langevin E., Zhu M., Bonaparte M., Machabert T., Savarino S., Zambrano B., Moureau A., Khromava A. (2018). Effect of Dengue Serostatus on Dengue Vaccine Safety and Efficacy. N. Engl. J. Med..

[187] Larson H.J., Hartigan-Go K., de Figueiredo A. (2019). Vaccine confidence plummets in the Philippines following dengue vaccine scare: Why it matters to pandemic preparedness. Hum. Vaccines Immunother..

[188] Huang C.Y.H., Kinney R.M., Livengood J.A., Bolling B., Arguello J.J., Luy B.E., Silengo S.J., Boroughs K.L., Stovall J.L., Kalanidhi A.P. (2013). Genetic and Phenotypic Characterization of Manufacturing Seeds for a Tetravalent Dengue Vaccine (DENVax). PLoS Negl. Trop. Dis..

[189] Higa Y. (2011). Dengue Vectors and their Spatial Distribution. Trop. Med. Health.

[190] Abreu F.V.S., Ribeiro I.P., Ferreira-de-Brito A., Santos A., Miranda R.M., Bonelly I.S., Neves M., Bersot M.I., Santos T.P.D., Gomes M.Q. (2019). Haemagogus leucocelaenus and Haemagogus janthinomys are the primary vectors in the major Yellow fever outbreak in Brazil, 2016–2018. Emerg. Microbes Infect..

[191] Williams C.R., Webb C.E., Higgs S., van den Hurk A.F. (2022). Japanese Encephalitis Virus Emergence in Australia: Public Health Importance and Implications for Future Surveillance. Vector Borne Zoonotic Dis..

[192] Williams P.C., Bartlett A.W., Howard-Jones A., McMullan B., Khatami A., Britton P.N., Marais B.J. (2021). Impact of climate change and biodiversity collapse on the global emergence and spread of infectious diseases. J. Paediatr. Child Health.

